# L-Carnitine Supplementation in Overweight Dogs: An Uncontrolled Pilot Study on Nutrient Digestibility, Metabolic Health, and Body Condition

**DOI:** 10.3390/vetsci13101046

**Published:** 2026-10-06

**Authors:** Oguzhan Kahraman, Zekeriya Safa Inanc, Tugba Melike Parlak, Metin Celik, Samed Damar, Burak Dik

**Affiliations:** 1 Department of Animal Nutrition and Nutritional Diseases, Faculty of Veterinary Medicine, Selcuk University, 42130 Konya, Türkiye; okahraman@selcuk.edu.tr (O.K.); samed.damar@selcuk.edu.tr (S.D.); 2 Department of Pharmacology and Toxicology, Faculty of Veterinary Medicine, Selcuk University, 42130 Konya, Türkiye; tugba.parlak@selcuk.edu.tr (T.M.P.); burakdik@selcuk.edu.tr (B.D.); 3 Department of Pharmacology and Toxicology, Faculty of Veterinary Medicine, Erciyes University, 38039 Kayseri, Türkiye; metincelik@erciyes.edu.tr

**Keywords:** digestibility, L-carnitine supplementation, fatty acid metabolism, antioxidant capacity, weight management, canine obesity

## Abstract

Background: L-carnitine supports mitochondrial fatty acid transport and has been proposed for weight management, but its short-term effects in overweight dogs are unclear. This pilot study aimed to generate preliminary, hypothesis-generating data on these effects, including possible dose–response patterns, to inform the design of a future randomized, placebo-controlled trial. Methods: Twenty-one Golden Retriever dogs (8–10 years) were assigned to three groups of seven: a control diet group (BCS < 7) receiving no supplementation, receiving no supplementation, and two overweight groups (BCS ≥ 7) receiving 1 g/day or 2 g/day oral L-carnitine for 30 days. Digestibility, faecal dry matter and consistency, BCS, body weight, appetite (4-day monadic feeding test), and serum biochemical, endocrinological and oxidative-status parameters were assessed. Results: BCS, as assessed by a blinded investigator, decreased from baseline at both doses (*p* < 0.05), more markedly at 2 g/day; however, body weight remained unchanged throughout the study period. Food intake during the post-supplementation appetite test was lower at 2 g/day than in the healthy reference group (*p* < 0.05), though no pre-supplementation baseline appetite measurement was available for comparison. Serum cholesterol decreased and total antioxidant status (TAS) increased at both doses by day 30 (*p* < 0.05); however, HDL also decreased at both doses and triglycerides increased at 2 g/day, indicating that lipid profile changes were not uniformly favourable. Insulin decreased at both doses, and visfatin decreased significantly at 2 g/day (*p* < 0.05). No significant changes in digestibility were observed. Conclusions: In this uncontrolled pilot study, short-term L-carnitine supplementation was associated with dose-related within-group changes in selected metabolic indicators in overweight dogs, without measurable weight loss within 30 days. Notably, the absence of uniformly favourable lipid profile changes and the lack of an untreated overweight control group limit interpretation. These findings should be regarded as hypothesis-generating and support the rationale for a future placebo-controlled trial, rather than establishing a definitive treatment effect, before L-carnitine can be recommended as an adjunct to longer-term canine obesity-management strategies.

## Simple Summary

Many pet dogs carry extra body fat, which can lead to health problems such as joint disease, diabetes-like changes, and a shorter lifespan. L-carnitine is a natural compound that helps the body turn fat into energy, and it is sometimes added to weight-control diets for pets. This study looked at what happens when overweight dogs are given L-carnitine by mouth every day for 30 days, at two amounts (1 g or 2 g per day), compared with dogs of normal body condition that received none. We measured the dogs’ body condition, how much they wanted to eat, how well they digested their food, and several blood markers related to fat and sugar metabolism. Dogs in the L-carnitine groups showed a lower body condition score and ate less during a four-day short-term voluntary food intake test conducted after 30 days, alongside favourable within-group changes in blood insulin, an antioxidant marker, and a hormone called visfatin. However, their body weight did not change significantly within the 30-day period, likely because the study was short. Importantly, because the study did not include an untreated overweight comparison group, these changes cannot be attributed to L-carnitine supplementation with certainty and should be interpreted as exploratory findings. These findings suggest that L-carnitine may be a useful, safe addition to weight-management programs for overweight dogs, particularly when combined with diet and exercise, and support further research into its longer-term use.

## 1. Introduction

Canine obesity is a significant health concern affecting 20–40% of the general dog population, with some studies reporting even higher rates of up to 59% [1,2]. Evidence from large-scale epidemiological studies suggests that the prevalence of excess body condition in dogs peaks during the mature life stage, with middle-aged and senior dogs at increased risk compared with younger adults, although this pattern varies by breed, sex, and neuter status [3,4]. Pets are particularly susceptible to weight gain when offered unrestricted access to highly palatable and calorie-rich diets. Among macronutrients, dietary fat provides the highest energy density and is the most efficiently stored form of energy in the body [5]. In canine studies, diets rich in fat but low in carbohydrates have been shown to promote greater fat accumulation and weight gain compared to those with lower fat and higher carbohydrate content. However, when consumed in excess, all macronutrients (including fats, proteins, and carbohydrates) can contribute to increased body fat [6].

Obesity in dogs is associated with numerous health issues, including metabolic disorders, endocrine diseases, reproductive problems, cardiopulmonary diseases, urinary disorders, dermatological conditions, and neoplasia [2]. While obesity is generally considered to occur when a dog’s body weight is at least 15% above ideal [1], there is considerable variation in how it is assessed and reported. Studies have used different methods to classify dogs as overweight or obese, including body condition score (BCS) charts, body fat index charts, and owner assessments [7]. Many studies indicate that dogs with a BCS ≥ 7, as well as those meeting the World Small Animal Veterinary Association (WSAVA) criteria with a BCS ≥ 6, are considered to be above their ideal body condition score and overweight [8,9,10,11].

L-carnitine is a conditionally essential amino acid-like compound that plays a crucial role in fatty acid metabolism. It is primarily involved in the transport of long-chain fatty acids into the mitochondria during the β-oxidation process [12]. L-carnitine’s slimming effect mechanism in weight loss is primarily attributed to its role in enhancing fatty acid oxidation and energy metabolism. Interestingly, the effectiveness of L-carnitine supplementation may be dose-dependent and influenced by individual factors: L-carnitine (250 mg) supplementation in Labrador Retrievers has been observed to improve exercise performance, reduce body fat mass, increase muscle mass, enhance antioxidant capacity, and decrease oxidative stress [13].

The management of obesity in dogs is a challenging aspect of routine veterinary practice. Furthermore, successful weight loss is often unsustainable in the long term. At this stage, it is crucial for veterinarians to educate pet owners that obesity is a serious medical condition and, if not appropriately treated, can lead to severe consequences. Additionally, identifying endocrinological and immunological changes in obese dogs holds significant importance [14]. During periods of calorie restriction and negative energy balance, L-carnitine supplementation may enhance the breakdown of dietary and endogenous fatty acids, thereby decreasing amino acid catabolism and preserving muscle tissue. Consequently, under both maintenance and deficit energy conditions, L-carnitine has the potential to positively influence body composition by reducing fat stores and supporting muscle mass retention or gain [15]. Lower concentrations of carnitine have been found in overweight dogs compared to lean dogs, and carnitine deficiency is associated with fat accumulation and altered lipid metabolism [13,16]. L-carnitine supplementation in obese or overweight cats has been associated with weight loss and less pronounced increases in plasma fatty acids and beta-hydroxybutyrate [17]. In obese dogs, successful weight loss has been associated with improved insulin sensitivity and reductions in circulating inflammatory adipokines [18]. In obese cats subjected to energy-restricted diets, L-carnitine supplementation has been variably reported to enhance caloric deficit [17], support weight loss [19], or show no significant effect [20].

L-carnitine treatment is limited in overweight dogs; the metabolic benefits observed in cats and humans suggest that L-carnitine supplementation could be a promising area for further research in obesity management in dogs. In short-term use, L-carnitine is expected to facilitate the conversion of fatty acids into energy, support fat oxidation, contribute to the preservation of muscle mass, accelerate metabolism, and exhibit effects that reduce oxidative stress. The majority of research on L-carnitine has focused on human participants, especially athletes; however, the findings across studies have been inconsistent [13].

To date, no published dose-ranging pilot study has simultaneously evaluated the effects of L-carnitine supplementation on nutrient digestibility, oxidative status, and adipokine profiles in elderly overweight dogs of a defined breed. In particular, the inclusion of visfatin—an adipokine increasingly recognised as a marker of adipose tissue dysfunction and insulin resistance in both humans and companion animals—as an outcome variable represents a novel aspect of the present investigation in the context of canine obesity research. Furthermore, whereas previous studies have largely relied on single-dose designs or have not reported digestibility data alongside metabolic endpoints, the present study prospectively characterises the response across two fixed doses under field conditions, thereby generating the preliminary effect-size and variance estimates required to power a future randomised, placebo-controlled trial. Taken together, these features distinguish the present work from the existing literature and underscore its hypothesis-generating purpose. Therefore, the aim of this pilot study was to generate preliminary, hypothesis-generating data on the digestibility, metabolic, and appetite-related effects of two oral L-carnitine doses in overweight dogs, in order to inform effect-size estimation and dose selection for a future randomized, placebo-controlled trial, rather than to establish a definitive treatment effect.

## 2. Materials and Methods

### 2.1. Experimental Design

This study was reviewed and approved by the Selcuk University, Veterinary Faculty, Experimental Animals Production and Research Center Ethics Committee (decision dated 2 May 2024, approval number 2024/073). The dogs were housed in individual kennels within the canine research unit of the University of Selcuk, Faculty of Veterinary Medicine, Prof. Dr. Humeyra Ozgen Research and Application Farm.

Twenty-one Golden Retriever dogs (aged 8–10 years) housed at the Selcuk University, Faculty of Veterinary Medicine, Prof. Dr. Humeyra Ozgen Research and Application Farm, were included in the study. Fourteen dogs were classified as overweight (BCS ≥ 7), whereas seven dogs had a normal body condition (BCS < 7). Obesity was diagnosed using a 1–9 body condition scoring system, as described by Chun et al. [8] and widely accepted for use in dogs. For dogs, the corresponding body condition score (BCS) for obesity is 8 or 9, while the ideal weight corresponds to 4 or 5 on the 9-point BCS scale [5].

The study included a total of 21 Golden Retriever breed dogs housed at the research unit in individual kennels. Fourteen of the dogs had a BCS of at least 7 and were considered overweight. These 14 dogs were divided into 2 groups of 7 dogs each, with similar gender, age, BCS, and body weights. The kennels had open (510 × 230 cm) and closed (190 × 190 cm) areas for dogs, including a bunk and free access to clean drinking water. The kennel’s climate temperature fluctuated from 15 to 22 °C.

L-carnitine supplementation was administered as fixed daily doses rather than body weight-adjusted doses. This dosing regimen was intentionally selected to reflect routine clinical practice in owned dogs, thereby enhancing the translational relevance and practical applicability of the findings.

Dogs were divided into three groups as follows:

**1.**  Basal diet (Control) group: Dogs were selected according to the World Small Animal Veterinary Association (WSAVA) 9-point body condition score (BCS) system, and only dogs with a BCS ≤ 6 were eligible for inclusion in the study. Seven eligible dogs were assigned to the basal diet (control) group and received no L-carnitine supplementation. The commercial extruded dry dog food used as the basal diet was provided daily, and the daily food allowance was individually calculated according to each dog’s metabolizable energy requirement (95 kcal ME/kg BW^0.75^/day) [21]. No L-carnitine, medication, or other supplemental feed additive was orally administered to dogs in this group throughout the entire 30-day study period, including the 4-day monadic feeding test.
**2.**  L-carnitine-1 g group (Basal diet + 1 g/day levocarnitine group): Seven dogs with a body condition score of 7 or higher were classified as overweight. These dogs received basal commercial dry dog food in quantities individually adjusted to meet their daily energy requirements. In addition to the basal diet, each dog was orally administered 1 g/day of levocarnitine (Metacartin 1 g/10 mL oral solution vial, World Medicine Pharmaceutical Industry and Trade Inc., Türkiye).
**3.**  L-carnitine-2 g group (Basal diet + 2 g/day levocarnitine group): This group also consisted of seven overweight dogs with a body condition score of ≥7. The dogs were maintained on the same basal commercial dry dog food, with the daily ration adjusted according to individual energy requirements. Each dog received same levocarnitine orally at a dose of 2 g/day.

L-carnitine supplementation was continued without interruption throughout the entire 30-day study period, including during the 4-day monadic feeding test. After the study groups were formed, the dogs were started on a diet of only commercial dry dog food for 30 days. The ingredient composition and analysed chemical composition of the commercial dry dog food used throughout the study are presented in Table 1.

The amount of food offered was consumed by all dogs without any refusal. Individually housed dogs, with little opportunity to move, may have daily energy requirements (DERs) as low as 70 kcal ME/kg^0.75^. All dogs were taken for a brisk walk for 1 h each day; therefore, the dogs’ metabolic energy requirements were calculated using the formula 95 kcal ME/kg^0.75^ [21]. Dry matter (DM), crude ash, ether extract (EE), crude fibre (CF), and crude protein (CP) levels of the dry dog food were analysed according to the methods of the Association of Official Agricultural Chemists [23]. The total dietary fibre (TDF) content of the dry dog food was analysed using an assay kit (Megazyme, Wicklow, Ireland) according to the procedure described by Kara [24]. The metabolisable energy level of the food was calculated using the following formulae, according to the guidelines of the National Research Council [22]:

*Gross energy (GE, *kcal/kg DM*) = (5.7 × CP) + (9.4 × EE) + [4.1 × (NFE + CF)]*

*Nitrogen-free extract (NFE, *%*) = DM − (EE + CP + Ash + CF)*

*Energy digestibility (*%*) = 91.2 − (1.43 × CF%)*

*Digestible energy (DE, *kcal/kg DM*) = GE × (Energy digestibility/100)*

*Metabolizable energy (ME, *kcal/kg DM*) = DE − (1.04 × CP)*

*Daily food allowance (*g/day*) = (Dog’s ME requirement × 1000)/Dietary ME (*kcal/kg*)*

Blood samples were collected from the V. cephalica antebrachii on days 0 and 30 of the study before feeding. The levocarnitine treatment trial lasted 30 days. On the last day of the study, dogs were weighed, and their body conditions were scored. Body condition scores at baseline and Day 30 were independently assessed by two investigators using the same 9-point BCS system. The two assessors performed their evaluations independently, and the mean of the two scores was used as the final BCS value for each dog.

This experiment was conducted as a non-terminal protocol; no animals were euthanised or relocated at any stage. All procedures were performed within the dogs’ original individual kennels at the institutional research facility to minimise stress and environmental disruption. Accordingly, the animals remained in their familiar housing conditions throughout the entire study period, including the post-experimental phase. No generative artificial intelligence (GenAI) tools were used to generate text, data, or graphics, or to assist in study design, data collection, analysis, or interpretation for this study.

### 2.2. Stool Consistency Scoring

Before collecting faeces in the last four days of the levocarnitine experiment, daily faecal consistency scoring was performed by three different authors (academic staff) of this study. The faecal consistency was scored according to a 1–5 scale as follows: 1, diarrhoea-like and watery; 2, soft and slightly shaped; 3, soft and shaped, leaving marks on the floor; 4, well-formed, undispersed; 5, very well-formed and dry [25].

### 2.3. Assessment of Levocarnitine’s Effect on Appetite

After day 30 of the research, an appetite test was administered for four days to assess the impact of levocarnitine on satiety. The monadic test (one-bowl test) is used to measure daily intake of dog food. Each dog was provided with 1 kg of food (exceeding its daily requirements). Dogs were allowed to consume food freely for 20 min. After the pre-weighed food was provided, the researcher moved away from the kennel to a location outside the dog’s visual field and remained out of sight throughout the 20 min test period. No verbal, visual, or physical interaction with the dog occurred during this period. At the end of 20 min, the researcher returned, removed the food bowl, and weighed the remaining food. After 20 min, the bowls were removed, and the remaining food was weighed to determine the amount consumed [26]. In this test, no food was refused, and consuming a small amount of food within 20 min was considered to indicate a decreased appetite in the dog. The 20 min test duration was selected as a standardized short-term feeding period for the assessment of voluntary food intake, consistent with a previously reported canine feeding protocol in which food intake was quantified over a 20 min period [27]. No baseline monadic voluntary food intake test was performed before L-carnitine supplementation. Therefore, voluntary food intake was evaluated only at the end of the supplementation period as a between-group comparison.

All dogs used in this study were institutional research animals that were maintained under standardized housing and feeding conditions during the study period, receiving controlled daily rations under this regimen. Consequently, they did not display uncontrolled food-seeking behaviour or excessive food consumption during routine feeding under these standardized conditions. The same appetite test protocol was applied to all experimental groups under identical environmental conditions. In this test, lower food consumption within the 20 min test period was interpreted as reduced voluntary food intake and increased satiety. To ensure unrestricted access to food and to allow accurate quantification of voluntary food intake and appetite, each dog was offered approximately 1 kg of the experimental diet, an amount exceeding the expected intake during the test period.

### 2.4. Assessment of the Dry Food’s Nutrient Digestibility Coefficients

Dogs were fed extruded dry dog food in a single meal daily. The trial lasted a total of 30 days; following 25 days of adaptation, total faeces were collected from each dog for five days to determine individual apparent nutrient digestibility. During the last 5 days of the trial, each dog’s faeces were collected and weighed daily. The faecal samples were stored in a freezer (−18 °C). On the day of analysis, the faecal samples were thawed and mixed at laboratory temperature (23–25 °C). The samples were then dried in an oven (VWR, Venti-Line, USA) at 55 °C for 48 h. The faecal samples and the experimental diet were analysed for dry matter (DM), crude protein (CP), ash (A), and crude fat (CF) content to determine the apparent total tract digestibility (ATTD) of nutrients [28]. In the digestibility calculation, which does not account for endogenous losses, the following formula was used [29]:

*ATTD (*%*) = [(*g *of nutrient intake − *g* of nutrient excretion)/*g* of nutrient intake] × 100*

### 2.5. Haematological, Biochemical, and Endocrinological Analyses

Haematological analyses (WBC, RBC, haematocrit, haemoglobin, platelets) were performed on whole blood samples in EDTA tubes using a haematology analyser (Mindray Bio-Medical Electronics, Shenzhen, China). Biochemical analyses (amylase, ALT, AST, total cholesterol, triglycerides, glucose, LDL, HDL) were conducted on serum samples in gel and clot activator tubes using a biochemical autoanalyser (BT-3000 Plus, Biotecnica Instruments, Rome, Italy).

Insulin (Canine Insulin, Cat. No. E0048Ca), glucagon (Canine Glucagon, Cat. No. E0108Ca), adiponectin (Canine Adiponectin, Cat. No. E0076Ca), irisin (Canine Irisin, Cat. No. E0460Ca), visfatin (Canine Visfatin, Cat. No. E0222Ca), and TBARS (Canine Thiobarbituric Acid Reactive Substances, Cat. No. E0132Ca) ELISA kits were obtained from Bioassay Technology Laboratory (Shanghai, China), and the TAS (Total Antioxidant Status, Cat. No. E-BC-K801-M) ELISA kit was obtained from Elabscience Biotechnology Inc. (Houston, TX, USA). Endocrinological (insulin, glucagon, adiponectin, irisin, visfatin) and oxidant/antioxidant parameters (TAS, TBARS) were measured using commercial ELISA kits, following the procedures specified by the manufacturer, and the readings were taken with an ELISA reader (Bio-Tek Instruments Inc., MWG Lambda Scan 200, Winooski, VT, USA).

### 2.6. Statistical Analysis

The sample size was determined a priori using G*Power software (version 3.1. Heinrich Heine University, Düsseldorf, Germany). The primary sample size calculation was based on inter-group comparisons among the three study groups using one-way ANOVA. Assuming a large effect size (Cohen’s f = 0.75), a significance level (α) of 0.05, and a statistical power (1 − β) of 0.80, the minimum required sample size was calculated as 21 dogs (7 per group). This sample size also provided >90% statistical power to detect within-group temporal changes using paired *t*-tests (Cohen’s d = 1.50, α = 0.05). Therefore, seven dogs were included in each group to ensure adequate statistical power for the primary outcome measures.

The data were statistically analysed using SPSS 25.0 software (SPSS, Inc., Chicago, IL, USA). The normality of the data was assessed using the Shapiro–Wilk test. Biochemical, endocrinological, and oxidative-status parameters were evaluated using the paired *t*-test. For the voluntary food intake assessment, intake recorded during the four consecutive 20 min test sessions was averaged for each dog, resulting in one individual mean value per dog. This mean intake was normalized to metabolic body weight using the body weight measured at the end of the supplementation period and expressed as g/kg BW^0.75^ per 20 min test. Normalized voluntary food intake was analysed using one-way analysis of variance (ANOVA), followed by Bonferroni-adjusted post hoc comparisons, with each dog considered the experimental unit (*n* = 7 per group). Digestibility parameters were analysed using one-way ANOVA followed by the Bonferroni post hoc test. Non-parametric data (body condition scores and faecal scores) were evaluated using the Kruskal–Wallis test and post hoc Dunn–Bonferroni tests. Statistical significance was set at *p* < 0.05. Parametric data are presented as mean ± standard deviation (SD), while non-parametric data are presented as median and interquartile range (IQR).

## 3. Results

**Figure 1:**
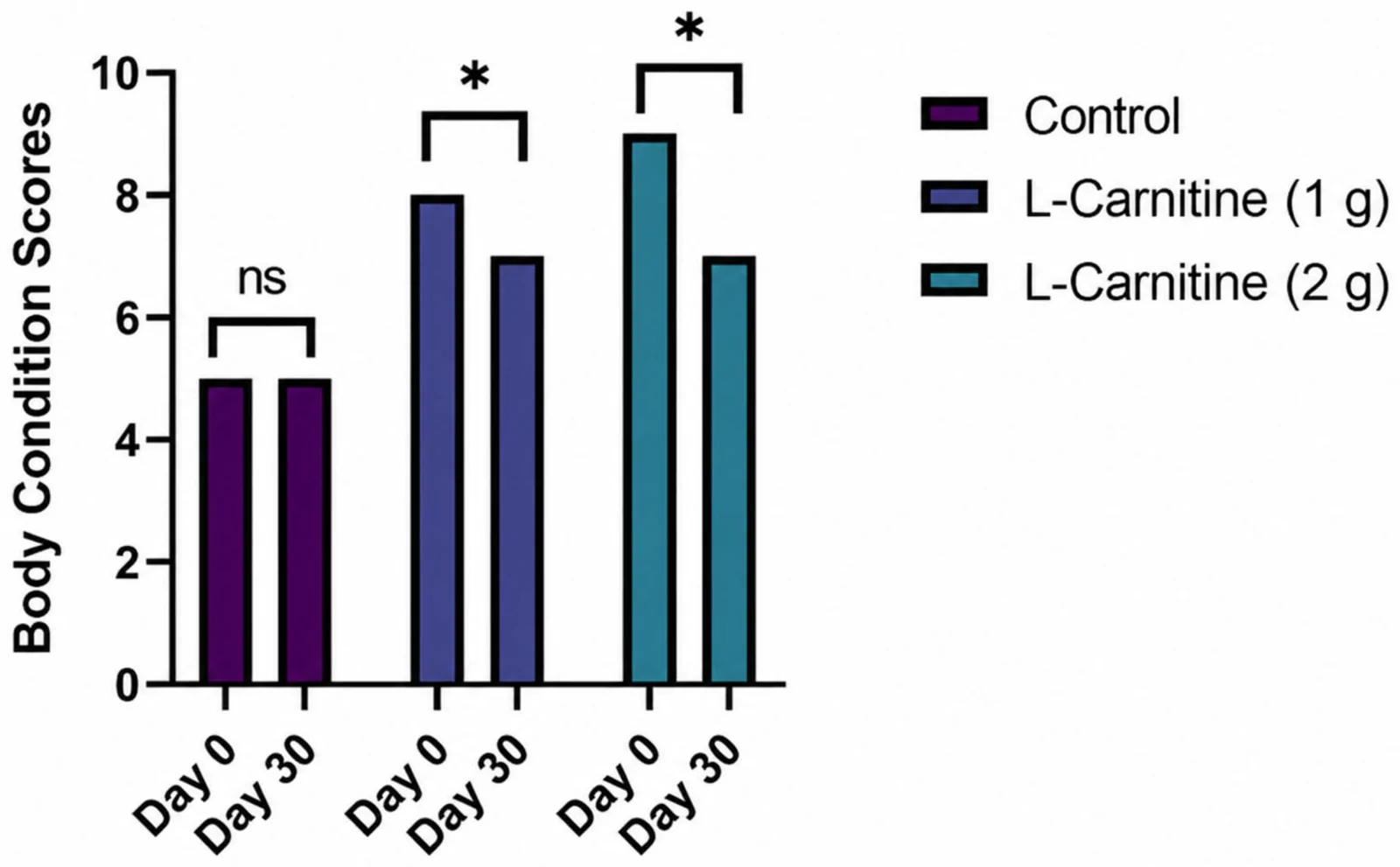
Pre- and post-supplementation changes in body condition scores in overweight dogs receiving L-carnitine (1 g and 2 g) dietary supplementation. The * symbol indicates a significant difference between two time points within the same group (*p* < 0.05), whereas ns indicates no significant difference (*p* > 0.05).

Compared to the body condition scores (BCS) before starting the study, BCS was lower at day 30 following L-carnitine supplementation (1 g and 2 g), with this decrease being more pronounced in the L-carnitine-2 g group (*p* < 0.05, Figure 1). Compared with the control group, food intake during the appetite test was lower in the L-carnitine groups (1 g and 2 g), but a statistically significant difference was found only in the L-carnitine-2 g group (*p* < 0.05).

**Figure 2:**
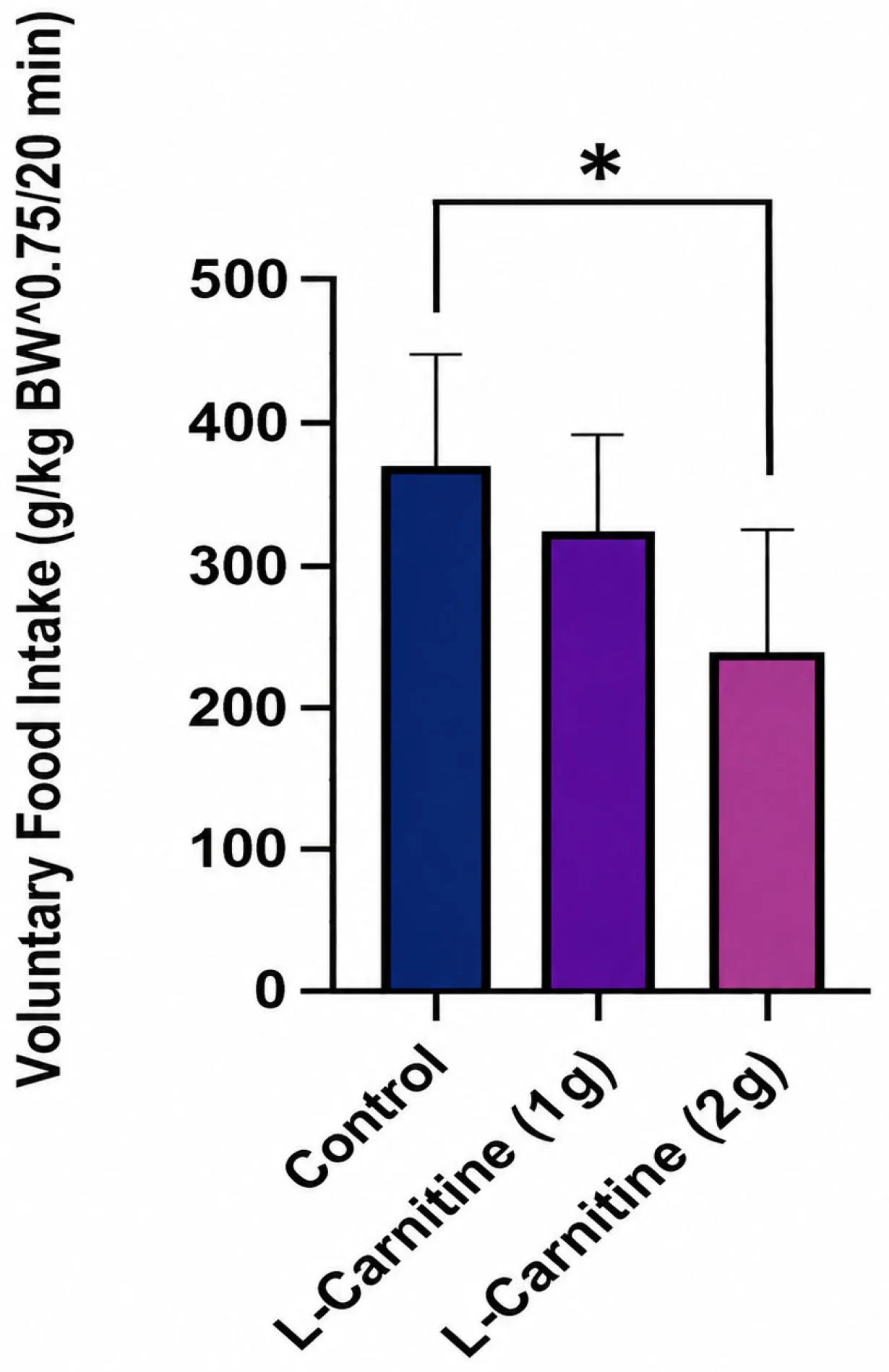
Mean voluntary food intake normalized to metabolic body weight (g/kg BW^0.75^ per 20 min test) during the four-day post-supplementation appetite assessment. Data are presented as mean ± SD (*n* = 7 dogs/group). * indicates a significant difference between the L-carnitine-2 g group and the control group (Bonferroni-adjusted *p* = 0.003). The other pairwise comparisons were not significant.

When voluntary food intake was normalized to metabolic body weight, mean intake was 29.14 ± 4.89, 24.27 ± 3.08, and 19.03 ± 5.97 g/kg BW^0.75^ per 20 min test in the control, L-carnitine-1 g, and L-carnitine-2 g groups, respectively. A significant overall difference among groups was detected (*p* = 0.004). Bonferroni-adjusted post hoc comparisons showed that normalized voluntary food intake was significantly lower in the L-carnitine-2 g group than in the control group (*p* = 0.003), whereas the differences between the control and L-carnitine-1 g groups (*p* = 0.220) and between the L-carnitine-1 g and L-carnitine-2 g groups (*p* = 0.168) were not significant (Figure 2).

**Figure 3:**
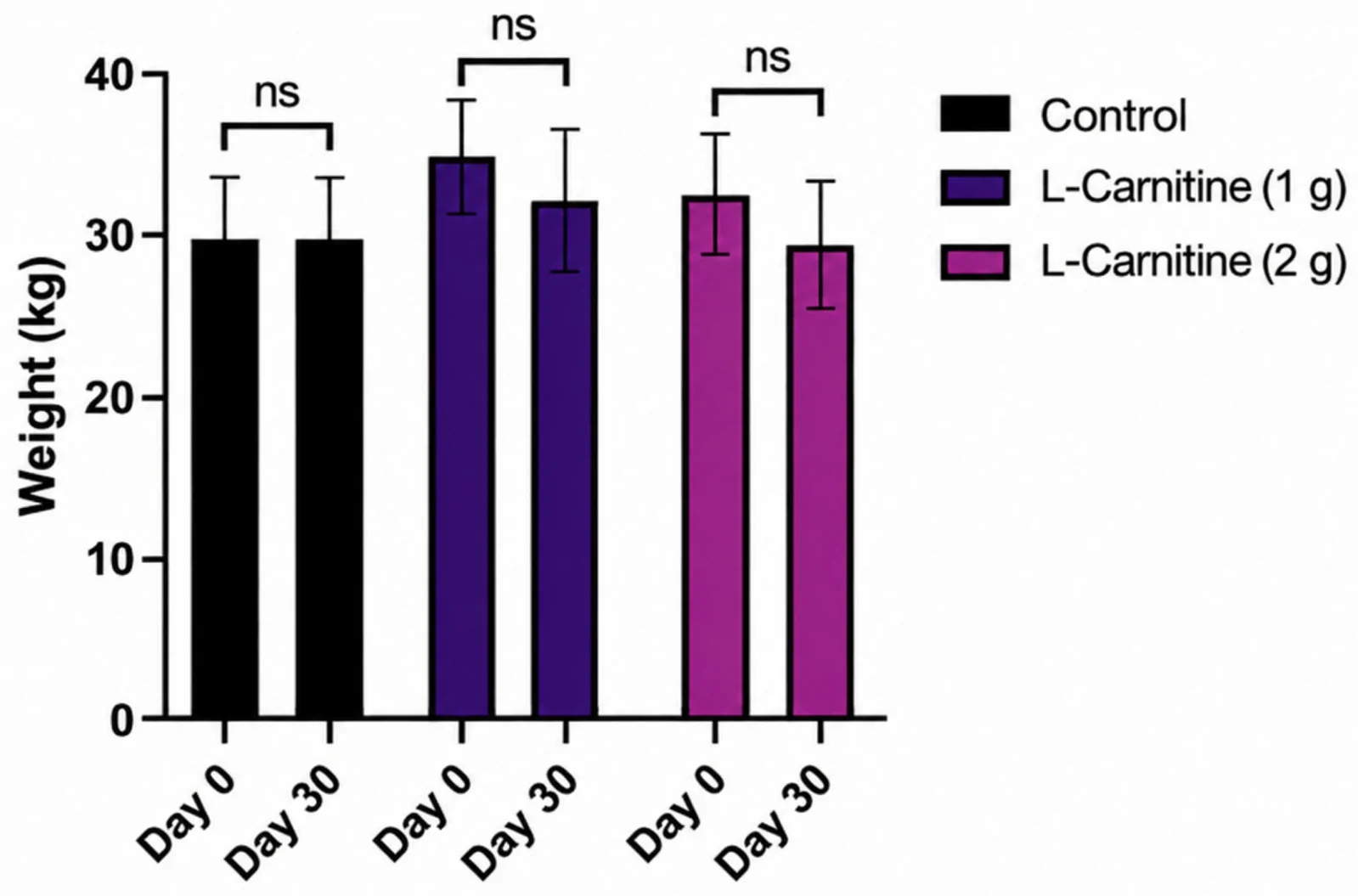
Pre- and post-supplementation changes in body weight in overweight dogs receiving L-carnitine (1 g and 2 g) dietary supplementation. ns indicates no significant difference between the two time points within the same group (*p* > 0.05).

Despite the decrease in appetite levels, no statistical difference in body weight was observed in either group (*p* > 0.05, Figure 3). Mean body weight in the Control group remained essentially unchanged, from 29.66 ± 3.36 kg at Day 0 to 29.63 ± 2.95 kg at Day 30 (*p* = 0.957). In the L-carnitine-1 g group, body weight decreased from 34.60 ± 3.21 kg to 31.96 ± 3.61 kg (*p* = 0.065), whereas in the L-carnitine-2 g group it decreased from 32.21 ± 3.16 kg to 29.24 ± 2.50 kg, although these changes did not reach statistical significance (*p* = 0.078).

It was observed that serum cholesterol and HDL concentrations significantly decreased in overweight dogs in both the L-carnitine (1 g) and L-carnitine (2 g) groups on day 30 compared with baseline (day 0) (*p* = 0.007 and *p* = 0.014, respectively), while triglyceride concentrations significantly increased only in the L-carnitine-2 g group (*p* = 0.004, Table 2). L-carnitine supplementation (at doses of 1 g and 2 g) in overweight dogs resulted in a significant increase in TAS levels on day 30 compared with day 0 (*p* < 0.001 and *p* = 0.019, respectively, Table 2).

No statistically significant changes in nutrient digestibility parameters and fecal consistency scores were observed in overweight dogs after L-carnitine (1 g and 2 g) supplementation (*p* > 0.05) (Table 3).

With respect to endocrinological parameters, insulin concentrations were lower at day 30 than at baseline (day 0) in both the 1 g/day and 2 g/day groups (*p* < 0.05; Table 4). Additionally, visfatin concentrations were significantly lower at day 30 compared with baseline in the 2 g/day group (*p* < 0.05; Table 4). Platelet counts in the 1 g/day group were lower at day 30 than at day 0 (*p* < 0.05); however, this decrease remained within the reference range reported for dogs, suggesting no clinically relevant haematological change (Table 5).

## 4. Discussion

Weight loss in overweight and obese dogs can be achieved through various dietary interventions and supplements, with significant impacts on health and quality of life [30]. Some supplements have demonstrated potential benefits for weight management in dogs. A diet containing coconut oil, L-carnitine, lipoic acid, lysine, leucine, and fibre was found to promote weight loss and improvements in BCS, possibly by enhancing energy metabolism [31]. Weight loss in obese dogs has been associated with numerous health benefits, including improved insulin sensitivity, decreased inflammatory markers, and potential improvements in renal function [18,32,33]. Importantly, successful weight loss programmes can significantly enhance dogs’ health-related quality of life, with improvements observed in vitality, emotional well-being, and pain scores [34,35].

Carnitine is an essential cofactor for the transport of long-chain fatty acids from the cytosol into the mitochondria of cells, where they undergo β-oxidation. Therefore, increasing the dietary intake of L-carnitine may enhance the oxidation of long-chain fatty acids [36]. In a study on Labrador Retrievers, plasma carnitine levels were found to be lower in overweight dogs compared with lean dogs [37]. After administering 125 mg of L-carnitine supplementation to Labrador Retrievers, serum L-carnitine levels increased, energy expenditure and protein turnover were enhanced, and muscle mass was shown to increase [38]. In cats, during the first 28 days of L-carnitine supplementation, weight loss ranged from 0.21 to 0.26 kg across different diet groups, while long-term weight loss increased to between 1.46 and 2.02 kg, with significant improvements in body condition, including an increase in lean body mass and a reduction in fat mass [20]. An 18-week L-carnitine supplementation regimen in cats resulted in progressively increasing weight loss over time, hypothesised to occur due to enhanced mitochondrial transport of fatty acids for oxidation and increased excretion of fatty acids via urine [17]. In the current study, L-carnitine supplementation at different fixed daily doses did not produce measurable body weight loss within the 30-day study period; however, it was associated with a reduction in BCS and decreased food consumption during the short-term appetite test, particularly in dogs receiving 2 g/day. Although fixed daily doses rather than body weight-adjusted doses were used, greater improvements across the monitored clinical and metabolic parameters were observed in the 2 g/day group under the present field conditions, suggesting a possible dose-dependent pattern that warrants further investigation. Given the relatively short duration of the study and the absence of an untreated overweight control group, these findings may represent early within-group changes in clinical and metabolic indicators rather than established treatment effects. Nevertheless, because objective body composition parameters, such as fat mass and lean body mass, were not assessed, the improvement in BCS should not be interpreted as definitive evidence of altered body condition. Notably, the reduction in BCS was not accompanied by a measurable reduction in body weight; therefore, the observed change in BCS should be interpreted cautiously, as its biological significance remains uncertain.

In the present study, serum cholesterol concentrations decreased following L-carnitine supplementation at both doses, whereas HDL concentrations decreased and triglyceride concentrations increased in dogs receiving 2 g/day. These findings differ from many previous studies reporting improvements in lipid profiles after long-term L-carnitine supplementation. However, previous studies have also shown that short-term L-carnitine supplementation may not reduce triglyceride concentrations, even when combined with dietary restriction, suggesting that its metabolic effects may depend on treatment duration and metabolic conditions [39]. Such discrepancies may be attributable to differences in treatment duration, age of the animals, dietary management, physical activity, baseline metabolic status, and study design. Notably, Hall and Jewell [36] also reported increased triglyceride concentrations following prolonged dietary L-carnitine supplementation in elderly dogs, suggesting that the metabolic response to L-carnitine may vary depending on physiological and experimental conditions. Therefore, although serum cholesterol concentrations were lower at day 30, the concomitant reduction in HDL and the increase in triglycerides observed at 2 g/day suggest that the lipid profile changes associated with L-carnitine supplementation were not uniformly favourable in the present uncontrolled pilot study. Moreover, the increase in triglyceride concentrations observed in the 2 g/day group may reflect a transient metabolic adaptation rather than a detrimental effect of L-carnitine itself. Because the supplementation period was relatively short and the study involved elderly obese dogs, lipid metabolism may not have fully adapted to enhanced fatty acid transport and oxidation. In addition, age-related alterations in lipid homeostasis and individual metabolic variability may have contributed to this response. As the present study did not evaluate the mechanisms underlying these changes, these explanations remain speculative and warrant further investigation.

L-carnitine supplementation has been shown to have beneficial effects on insulin sensitivity and visfatin levels in various studies. L-carnitine administration, when combined with a hypocaloric diet, significantly decreased plasma insulin levels and HOMA-IR in patients with impaired fasting glucose or type 2 diabetes [40]. Interestingly, while L-carnitine appears to decrease insulin levels, visfatin, an adipocytokine with insulin-mimetic properties, shows a more complex relationship with insulin and glucose levels [41]. Visfatin levels have been reported to be higher in patients with type 2 diabetes than in healthy controls [42]. Furthermore, hyperglycaemia has been shown to increase circulating visfatin concentrations in healthy humans, while this glucose-induced increase was prevented by co-infusion of insulin or somatostatin [43]. Taken together, L-carnitine supplementation has been shown to improve insulin sensitivity and reduce insulin levels in patients with metabolic disorders. Although the evidence remains limited and is largely derived from human studies, the relationship between insulin, glucose, and visfatin suggests that the insulin-lowering effects of L-carnitine could indirectly influence visfatin levels. Consistent with this mechanism, a decrease in insulin and visfatin levels was observed in parallel with the dose of L-carnitine in the current study. These results are consistent with a possible insulin-sensitizing effect of the 2 g/day dose. However, insulin resistance was not directly quantified (e.g., HOMA-IR), and in the absence of an untreated overweight control group this interpretation remains speculative.

Studies have shown that L-carnitine is not only a lipid carrier but also an antioxidant [44,45]. It has been reported that L-carnitine inhibits the formation of reactive oxygen species through its iron ion metal-chelating ability and stabilises free radicals formed on the α-carbon through conjugation [45,46]. In patients receiving a daily dose of 1000 mg, a decrease in malondialdehyde (MDA) levels and an increase in the levels of antioxidant enzymes such as catalase (CAT), superoxide dismutase (SOD), and glutathione peroxidase (GPx) have also been observed [47]. In the current study, although not statistically significant, the TBARS level, an indicator of oxidative status, showed a decline, while total antioxidant status (TAS) significantly increased in both groups. This increase over the short period of 30 days may have contributed to the reduction in cholesterol, insulin, and visfatin levels. Furthermore, L-carnitine may have contributed to the more effective functioning of insulin by reducing cellular inflammation through its antioxidant properties and increasing insulin sensitivity in cells. It is possible that the continuing increase in TAS levels with longer-term treatment could help prevent cardiovascular disorders.

Carnitine palmitoyltransferase (CPT-1) is the rate-limiting step of the fatty acid oxidation pathway and a target for obesity treatment. Pharmacological stimulation of brain CPT-1 has been reported to reduce food intake and body weight [48]. It has been reported that L-carnitine supplementation in cats leads to increased energy expenditure and play motivation with energy expenditure [49]. In the current study, a reduction in appetite was observed in parallel with the dose of L-carnitine. The 2 g/day dose was associated with lower food consumption during the 20 min voluntary food intake tests conducted over four consecutive days. This finding is limited to short-term feeding behaviour during the four-day test period and should not be interpreted as evidence of sustained appetite suppression, as satiety-related hormones were not measured.

The haematological adverse effects of long-term drug or supplement use should be identified [50]. No adverse effects were noted at doses of 1 or 2 g of L-carnitine in the current investigation when assessing its effects on haemogram parameters and digestibility. Platelet levels were found to fall slightly with only the 1 g dose; however, this decline remained within the reference range for dogs reported in the literature [51].

## 5. Conclusions

As a preliminary, hypothesis-generating pilot study, this work was not designed or powered to establish a definitive causal effect of L-carnitine supplementation. Rather, its principal contribution is to provide effect-size and variability estimates that can inform the design—including dose selection and sample size calculation—of a future randomized, placebo-controlled trial.

In the absence of an untreated overweight control group, it is not possible to disentangle a specific pharmacological effect of L-carnitine from the influence of standardized housing, diet, and controlled exercise, which were common to all dogs throughout the study period.

The observed decreases in cholesterol concentrations and food intake during the appetite test are consistent with metabolic changes that, if sustained and confirmed in a controlled setting, may warrant further investigation in the context of longer-term metabolic health outcomes. These outcomes align with the known roles of L-carnitine in fatty acid oxidation, antioxidant activity, and appetite regulation. Furthermore, the absence of significant adverse effects highlights its safety for short-term use.

Future research should focus on the long-term effects of L-carnitine supplementation combined with dietary restriction and physical activity to better evaluate its potential in managing canine obesity. Identifying optimal dosages and investigating their broader impacts on metabolic and cardiovascular health will be critical for developing effective treatment protocols for overweight and obese dogs. The neuroprotective effects of L-carnitine against diseases such as dementia and Alzheimer’s disease suggest that it may also be beneficial for elderly overweight dogs.

**Limitations**

- An important limitation of the present study is the absence of an untreated overweight control group. Consequently, although significant improvements were observed following L-carnitine supplementation, the potential influence of standardized dietary management and lifestyle modifications cannot be completely excluded. Nevertheless, because each dog served as its own baseline control, inter-individual variability was minimized, allowing longitudinal evaluation of the clinical and metabolic changes associated with L-carnitine supplementation under field conditions. Furthermore, because no untreated overweight group was followed in parallel, none of the within-group changes reported in BCS, appetite, lipid profile, insulin, visfatin, or TAS can be attributed to L-carnitine independently of the standardized diet, controlled daily exercise, consistent housing, and the mere passage of 30 days, nor can regression to the mean be excluded. Every result in this manuscript should be read as a within-group, pre–post observation rather than as a demonstrated treatment effect. Future randomized placebo-controlled studies are warranted to further confirm these findings.
- The present study is that body composition was evaluated using BCS alone. Although BCS is a well-established and clinically relevant method for assessing adiposity in dogs, no direct measurements of fat mass, lean body mass, muscle condition score, or morphometric parameters were performed. Therefore, while the improvement in BCS suggests a favorable change in body condition, definitive conclusions regarding alterations in body condition cannot be drawn. Future studies incorporating objective body condition assessment techniques are needed to validate these findings.
- Another limitation of the present study is that voluntary food intake was not assessed using the same standardized monadic feeding protocol at baseline. Therefore, changes in voluntary food intake from Day 0 to Day 30 could not be determined, and the appetite findings should be interpreted only as end-of-study between-group differences. Future studies should include identical baseline and post-intervention voluntary food intake assessments to permit longitudinal evaluation of appetite-related responses.
- The use of fixed daily doses rather than body weight-adjusted doses resulted in variable mg/kg L-carnitine exposure across individual dogs within each group (1 g/day group: 26.1–32.4 mg/kg/day; 2 g/day group: 55.4–69.7 mg/kg/day). Although both dose levels are considered to fall within the safe range reported for dogs, this variability may have introduced individual-level heterogeneity in biological response that cannot be accounted for in the present analysis. Weight-adjusted dosing is therefore recommended for the future randomised, placebo-controlled trial.

## Figures and Tables

**Table 1:** Ingredients and chemical composition of the commercial dry dog food.

Ingredient	%
Chicken meal	22
Barley	26
Corn	10
Corn gluten	10
Rice	26.6
Potassium chloride	0.5
Salt	0.1
Vitamin/mineral mix ^1^	0.4
Sunflower oil	2.5
Beef tallow	1.9
**Chemical composition**	
Parameter	Value
Dry matter, %	91.5
Crude ash, %	5.28
Acid hydrolyzed ether extract, %	9.21
Crude fibre, %	6.45
Crude protein, %	18.45
Metabolizable energy (ME), kcal/kg ^2^	3311.1
Total dietary fibre, %	12.54
Calcium, %	1.3
Phosphorus, %	0.85
Sodium, %	0.35
Vitamin A, IU/kg	12,000
Vitamin D, IU/kg	1100
Vitamin E, mg/kg	60

^1^ Vitamin–mineral mixture (mg/kg): biotin 250, folate 2000, niacin 50,000, pantothenic acid 20,000, riboflavin 8000, thiamine 4000, vitamin B6 5000, vitamin B12 25, vitamin A 3600, cholecalciferol 125, vitamin E 50,000, manganese proteinate 70,000, zinc proteinate 100,000, iron proteinate 70,000, copper proteinate 14,000, iodine 1000, cobalt proteinate 350, selenium proteinate 140, molybdenum proteinate 700, magnesium oxide 35,000. ^2^ Determined using NRC [22] equations.

**Table 2:** Pre- and post-supplementation changes on biochemical parameters in overweight dogs receiving L-carnitine (1 g and 2 g) dietary supplementation (mean ± SD).

Parameter	Control (Day 0)	Control (Day 30)	L-Carnitine-1 g (Day 0)	L-Carnitine-1 g (Day 30)	L-Carnitine-2 g (Day 0)	L-Carnitine-2 g (Day 30)
Glucose (mg/dL)	100.85 ± 5.87	89.71 ± 13.53	95.71 ± 8.13	69.42 ± 6.24	98.14 ± 8.66	73.14 ± 8.82
Cholesterol (mg/dL)	230.71 ± 38.01	204.71 ± 60.59	269.00 ± 60.78	214.42 ± 65.55 *	260.85 ± 35.26	227.28 ± 50.90 *
Triglyceride (mg/dL)	43.71 ± 8.19	50.16 ± 6.91	51.00 ± 10.58	61.33 ± 22.74	45.42 ± 12.16	57.00 ± 14.49 *
HDL (mg/dL)	162.84 ± 21.00	144.40 ± 31.45	174.82 ± 30.70	146.77 ± 34.89 *	177.76 ± 18.81	159.58 ± 28.33 *
LDL (mg/dL)	19.57 ± 6.16	21.71 ± 11.36	30.71 ± 10.87	27.14 ± 13.54	25.71 ± 6.77	23.85 ± 9.63
Amylase (U/L)	817.00 ± 262.71	787.28 ± 276.70	985.85 ± 261.33	959.14 ± 222.47	971.28 ± 268.40	935.42 ± 268.28
TAS (mmol)	1.14 ± 0.18	1.11 ± 0.25	0.73 ± 0.18	1.49 ± 0.12 *	0.92 ± 0.24	1.48 ± 0.25 *
TBARS (nmol/mL)	13.35 ± 7.66	12.34 ± 8.09	22.07 ± 12.22	16.50 ± 8.97	13.16 ± 4.86	9.57 ± 2.60

HDL, high-density lipoprotein; LDL, low-density lipoprotein; TAS, total antioxidant status; TBARS, thiobarbituric acid reactive substances. * Significantly different from day 0 within the same group (*p* < 0.05).

**Table 3:** Pre- and post-supplementation changes on digestibility parameters in overweight dogs receiving L-carnitine (1 g and 2 g) dietary supplementation (mean ± SD).

Parameter	Control	L-Carnitine-1 g	L-Carnitine-2 g
DMD, %	79.55 ± 0.95	79.93 ± 2.11	80.06 ± 1.58
OMD, %	82.08 ± 1.18	82.96 ± 1.92	83.51 ± 1.36
EED, %	90.79 ± 0.67	91.46 ± 0.55	91.26 ± 0.66
CPD, %	79.49 ± 1.05	81.05 ± 1.15	80.78 ± 0.99
Faecal DM, %	30.64 ± 0.99	32.17 ± 2.61	32.17 ± 1.92
Faecal consistency score, median (IQR)	4.0 (0.63)	3.75 (0.63)	3.75 (0.5)

DMD, dry matter digestibility; OMD, organic matter digestibility; EED, ether extract digestibility; CPD, crude protein digestibility; DM, dry matter.

**Table 4:** Pre- and post-supplementation changes on endocrinological parameters in overweight dogs receiving L-carnitine (1 g and 2 g) dietary supplementation (mean ± SD).

Parameter	Control (Day 0)	Control (Day 30)	L-Carnitine-1 g (Day 0)	L-Carnitine-1 g (Day 30)	L-Carnitine-2 g (Day 0)	L-Carnitine-2 g (Day 30)
Glucagon (ng/mL)	10.70 ± 3.36	10.38 ± 5.45	13.57 ± 5.02	12.78 ± 5.14	10.05 ± 3.02	10.15 ± 2.64
Insulin (mIU/L)	5.44 ± 2.83	4.41 ± 2.65	7.98 ± 3.56	6.73 ± 4.59 *	4.91 ± 1.04	3.83 ± 1.60 *
Irisin (ng/mL)	35.40 ± 12.67	28.47 ± 17.25	48.50 ± 16.24	32.20 ± 15.25	34.56 ± 6.20	25.17 ± 5.93
Adiponectin (mg/L)	8.59 ± 2.21	7.94 ± 4.06	10.97 ± 2.91	7.98 ± 3.24	7.93 ± 1.51	6.77 ± 1.11
Visfatin (ng/mL)	22.08 ± 7.94	22.08 ± 10.73	29.04 ± 9.98	29.98 ± 13.98	21.63 ± 4.69	18.34 ± 3.47 *

* Significantly different from day 0 within the same group (*p* < 0.05).

**Table 5:** Pre- and post-supplementation changes on hematological parameters in overweight dogs receiving L-carnitine (1 g and 2 g) dietary supplementation (mean ± SD).

Parameters/Groups	Control (Day 0)	Control (Day 30)	L-Carnitine-1 g (Day 0)	L-Carnitine-1 g (Day 30)	L-Carnitine-2 g (Day 0)	L-Carnitine-2 g (Day 30)
WBC (10^3^/μL)	7.00 ± 1.77	7.04 ± 0.77	8.56 ± 3.32	7.69 ± 2.17	5.99 ± 1.34	7.18 ± 1.30
RBC (10^6^/μL)	6.78 ± 0.43	6.38 ± 0.63	6.65 ± 0.78	6.34 ± 0.66	6.91 ± 0.55	5.83 ± 1.48
HCT (%)	46.55 ± 2.80	43.55 ± 4.19	45.40 ± 5.85	43.67 ± 3.92	46.51 ± 4.31	39.31 ± 10.50
Hb (g/dL)	16.50 ± 1.00	15.82 ± 1.56	15.78 ± 1.62	15.57 ± 1.65	16.25 ± 1.43	14.27 ± 3.84
PLT (10^3^/μL)	339.14 ± 75.54	302.57 ± 79.38	360.14 ± 97.84	322.14 ± 85.66 *	296.42 ± 45.18	318.14 ± 97.70

* Significantly different from day 0 within the same group (*p* < 0.05).

## Data Availability

The original contributions presented in this study are included in the article. Further inquiries can be directed to the corresponding author(s).
